# Supplementary material for: The interactome of intact mitochondria by cross-linking mass spectrometry provides evidence for coexisting respiratory supercomplexes
Source: Mol Cell Proteomics. 2017 Dec 8;17(2):216–32. doi: 10.1074/mcp.RA117.000470 (PMC5795388; doi:10.1074/mcp.RA117.000470)
Supplement: Supplemental Data [file supp_RA117.000470_133922_0_supp_23978_fzffwf.zip › spectra_annotation/mito_spectra_annotation/376-1-10-1-14-1.pdf]

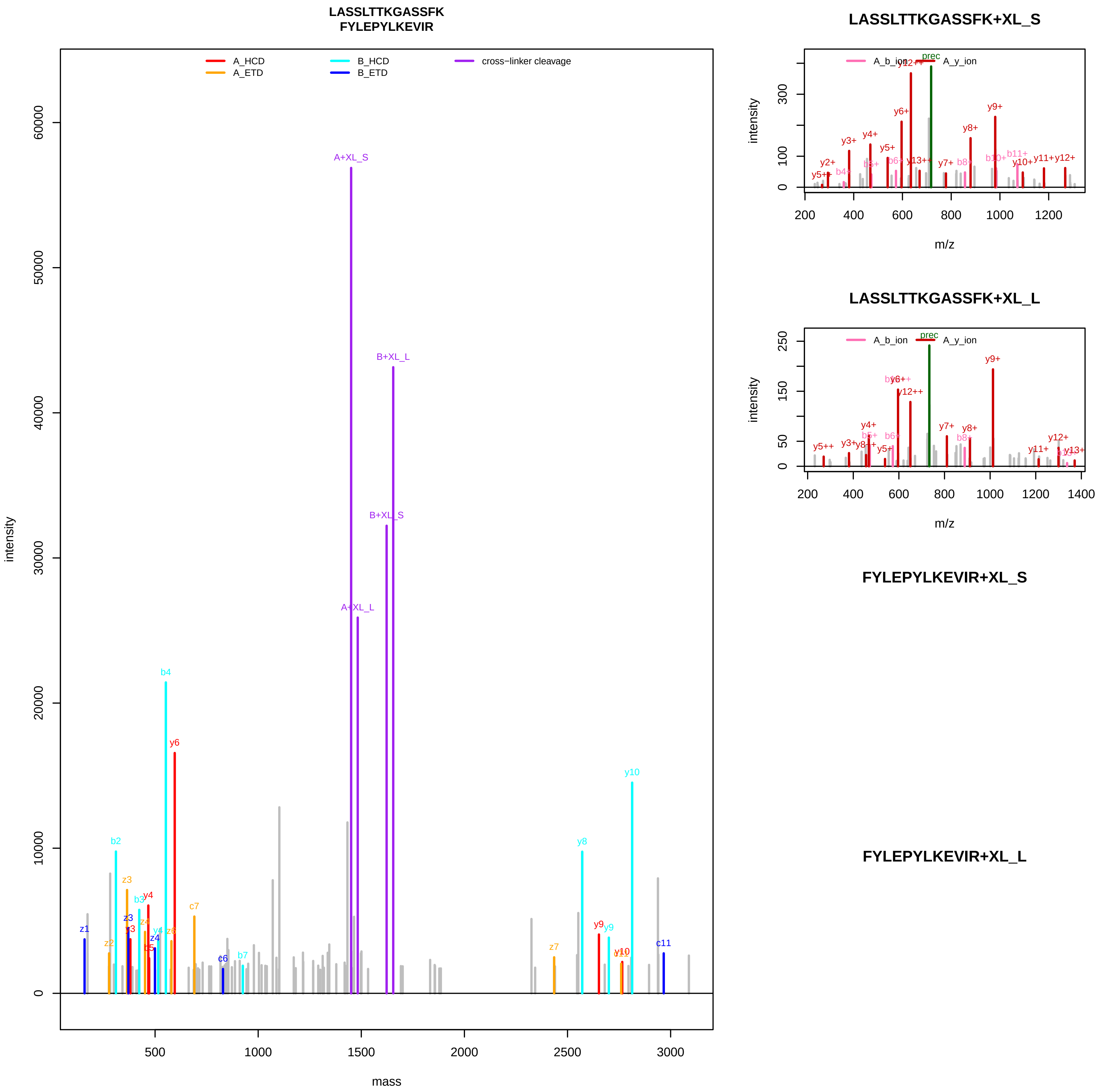

LASSLTTKGASSFK
FYLEPYLKEVIR
A_HCD
A_ETD
B_HCD
B_ETD
cross−linker cleavage
A+XL_S
B+XL_L
B+XL_S
A+XL_L
b4
y6
b2
z3
b3
y4
z1
z2
c7
c6
b7
y10
y8
y9
z7
c11
intensity
mass
0
10000
20000
30000
40000
50000
60000
500
1000
1500
2000
2500
3000
LASSLTTKGASSFK+XL_S
A_b_ion
A_y_ion
prec
y12++
y6+
y9+
y8+
y4+
y3+
y5+
y2+
y5++
y13++
y7+
y10+
y11+
y12+
b11+
b10+
b8+
b6+
b4+
m/z
200
400
600
800
1000
1200
0
100
300
LASSLTTKGASSFK+XL_L
y9+
y12++
y4+
y7+
y8+
y3+
y5++
y5+
y11+
y12+
y13+
b8+
b5+
b6+
150
250
1400
FYLEPYLKEVIR+XL_S
FYLEPYLKEVIR+XL_L
